# Antimicrobial Effect of *Thymus capitatus* and *Citrus limon* var. *pompia* as Raw Extracts and Nanovesicles

**DOI:** 10.3390/pharmaceutics11050234

**Published:** 2019-05-14

**Authors:** Roberto Pinna, Enrica Filigheddu, Claudia Juliano, Alessandra Palmieri, Maria Manconi, Guy D’hallewin, Giacomo Petretto, Margherita Maioli, Carla Caddeo, Maria Letizia Manca, Giuliana Solinas, Antonella Bortone, Vincenzo Campanella, Egle Milia

**Affiliations:** 1 Department of Biomedical Sciences, University of Sassari, 07100 Sassari, Italy; caesareus83@yahoo.it (R.P.); loran23@hotmail.it (E.F.); mmaioli@uniss.it (M.M.); gsolinas@uniss.it (G.S.); 2 Department of Chemistry and Pharmacy, University of Sassari, 07100 Sassari, Italy; julianoc@uniss.it (C.J.); gpetretto@uniss.it (G.P.); 3 Department of Medicine, Surgery and Experimental Science, University of Sassari, 07100 Sassari, Italy; luca@uniss.it; 4 Department of Life and Environmental Sciences, University of Cagliari, 09124 Cagliari, Italy; manconi@unica.it (M.M.); caddeoc@unica.it (C.C.); mlmanca@unica.it (M.L.M.); 5 Institute of Science of Food Production UOS Sassari-CNR, 07040 Sassari, Italy; guy.dhallewin@gmail.com; 6 Laboratory of Molecular Biology and Stem Cell Engineering, National Institute of Biostructures and Biosystems, 40129 Bologna, Italy; 7 Dental Unite, Azienda Ospedaliero-Universitaria, 07100 Sassari, Italy; nennebortone@tiscali.it; 8 Department of Clinical and Translational Medicine, Tor Vergata University of Rome, 00133 Rome, Italy; vincenzo.campanella@uniroma2.it

**Keywords:** Oral antimicrobials, caries prevention, natural extracts, nanovesicles

## Abstract

In view of the increasing interest in natural antimicrobial molecules, this study screened the ability of *Thymus capitatus* (TC) essential oil and *Citrus limon* var. *pompia* (CLP) extract as raw extracts or incorporated in vesicular nanocarriers against *Streptococcus mutans* and *Candida albicans*. After fingerprint, TC or CLP were mixed with lecithin and water to produce liposomes, or different ratios of water/glycerol or water/propylene glycol (PG) to produce glycerosomes and penetration enhancer vesicles (PEVs), respectively. Neither the raw extracts nor the nanovesicles showed cytotoxicity against human gingival fibroblasts at all the concentrations tested (1, 10, 100 μg/mL). The disc diffusion method, MIC-MBC/MFC, time-kill assay, and transmission electron microscopy (TEM) demonstrated the highest antimicrobial potential of TC against *S. mutans* and *C. albicans*. The very high presence of the phenol, carvacrol, in TC (90.1%) could explain the lethal effect against the yeast, killing up to 70% of *Candida* and not just arresting its growth. CLP, rich in polyphenols, acted in a similar way to TC in reducing *S. mutans*, while the data showed a fungistatic rather than a fungicidal activity. The phospholipid vesicles behaved similarly, suggesting that the transported extract was not the only factor to be considered in the outcomes, but also their components had an important role. Even if other investigations are necessary, TC and CLP incorporated in nanocarriers could be a promising and safe antimicrobial in caries prevention.

## 1. Introduction

As proposed in the “ecological plaque hypothesis”, a carious lesion develops under changed environmental conditions, producing a corresponding disturbance to the stability of the resident microflora [1]. Changes occur when the frequency of fermentable dietary carbohydrate intake is increased. In such a situation, the biofilm spends more time at low pH, allowing *Streptococcus mutans* to proliferate at the expense of the resident community. *S. mutans* contributes to the lowering of the pH and the growth of acidogenic species at the expense of the resident microflora [2,3]. This breakdown disrupts the de- and remineralization in teeth, pushing the equilibrium toward demineralization and tooth cavities. *Candida albicans* can appear in cariogenic biofilm when bonded to *S. mutans*, thus increasing the virulence of the biofilm and resulting in severe and recurrent caries in early childhood [4] and in salivary disorders [5].

In addition to fluorine, the capacity of which has been widely recognized as an anti-carious agent [5,6,7], a broad range of chemical agents have been used to reduce the cariogenicity of *S. mutans* in the biofilm [8]. Nevertheless, the increasing phenomenon of antibiotic resistance [9] necessitates the study of alternative therapeutic strategies to chemicals, and new substances, including natural extracts, have been widely investigated [10,11]. The antimicrobial capacity of biomolecules relies on the presence of chemical groups with distinct biosynthetic origins: Phenolic and polyphenols, terpenoids, alkaloids, lectins and polypeptides, and polyacetylenes [12,13]. In particular, terpenoids and flavonoids have demonstrated great potential against cariogenic bacteria and *C. albicans* [14,15,16,17]. Recently, encapsulation of natural molecules within lipid-based carriers has shown to increase their efficacy by protecting them from degradation [18], enhancing bioavailability [19]**,** and delivering adequate concentration to the target tissues [20] or microorganisms [21].

Within lipid-based carriers, lamellar vesicles are composed of phospholipids, drugs, and, in some cases, additive components, such as surfactants and co-solvents. The identification of the appropriate combination of vesicle components is a matter of intensive study. Phospholipid nanovesicles having high concentrations of glycerol as co-solvent (glycerosomes), and penetration enhancer-containing vesicles (PEVs) have demonstrated a high efficacy in carrying polyphenols. Indeed, PEVs were able to promote the penetration of the active components of *S. insularis* essential oil in the skin, greater than that of control liposomes, and similarly, glycerosomes potentiated the aptitude of the pompia extract to counteract oxidative stress in both keratinocytes and fibroblasts, preventing their death [22,23]. Their versatility and absence of toxicity can make them suitable carriers for the delivery of antimicrobial molecules.

Among the enormous number of plant genera, various types of *Thymus* of the Lamiaceae family, and *Citrus* of the Rutaceae family have attracted attention for their bactericidal, fungicidal, and anti-inflammatory activities [14,16,24]. In this work, *Thymus capitatus* (TC) and *Citrus limon* var. *pompia* (CLP), typical species of Sardinia (Italy), were investigated. A great concentration of phenols has been identified in the essential oil of TC [25]. In addition, the high availability of flavonoids rich in polyphenolic rings [23] suggest great antimicrobial capacity for CLP.

Thus, in view of the increasing general interest in developing antimicrobials of natural origins, which could, more specifically, be useful in caries prevention, this study aimed to assess the antimicrobial efficacy of TC essential oil and CLP extract used as raw extracts or incorporated in vesicular nanocarriers in the neutralization of *S. mutans* and *C. albicans*.

## 2. Materials and Methods

### 2.1. Plant Collection

The aerial parts (flowers, leaves and stems) of TC were collected in North Sardinia (40°82′02″ N; 8°63′10″ E) from wild plants growing on marl soil over limestone. Harvesting took place in mid-June when the plants were fully flowering, in the so-called “balsamic time”, which is the period of the plant growth cycle in which essential oil and/or specific metabolite production reaches its maximum.

CLP fruit was harvested in January from plants growing on alluvial soil located near Cedrino river in Orosei (40°37′75″ N; 9°71′23″ E). After harvest, the TC and CLP were immediately stored at 5 °C and processed within the following 24 h, as previously reported [23].

### 2.2. Extraction Procedures

The essential oil was extracted from the fresh aerial parts of TC. The collected parts were put into a heated (30 °C) ventilated armoire (Memmert 260, Spinea, Venice, Italy) until dry. Then, 150 g of the dried parts were powdered by milling the matrix with a ball-mill (Retsh Emax, Retsch GmbH, Haan, Germany) for 20 min, and steam-distilled for 2 h using a circulatory Clevenger-type apparatus (Merk KGaA, Dormstadt, Germany) according to the European Pharmacopoeia. The distillate was then dried over anhydrous sodium sulphate and the obtained essential oil was stored at low temperature until use. The chemical characterization of TC was conducted using a solution of the essential oil in hexane.

CLP fruit rind (flavedo and albedo) was removed within 24 h of harvest and subjected to extraction as previously reported [23]. Briefly, the rind was minced with a knife, dried, powdered, and dispersed in a water/ethanol (50:50 *v*/*v*) blend. After centrifugation, the supernatant was collected, ethanol was removed with a rotovapor (Buchi R 300, Buchi, Cornaredo, Italy), while water was removed by freeze-drying. A fine yellow powder was obtained, and vacuum stored until use. The chemical characterization of the extract was conducted using the powder dissolved in methanol (1:100 *w*/*v*).

### 2.3. Identification of TC and CLP Components

#### 2.3.1. GC-MS Analysis of TC Essential Oil

The GC-MS analysis of a TC essential oil solution in hexane (dilution ratio 1:200 *v*/*v*, injection volume 1 μL) was carried out by using an Agilent 7890 GC (Palo Alto, CA, USA) equipped with a Gerstel MPS autosampler, coupled with an Agilent 7000C MSD detector (Palo Alto, CA, USA). The chromatographic separation was performed on a HP-5MS capillary column (30 m × 0.25 mm, film thickness of 0.17 μm), and the following temperature program was used: 60 °C hold for 3 min, increased to 210 °C at a rate of 4 °C/min, held at 210 °C for 15 min, increased to 300 °C at a rate of 10 °C/min, and finally held at 300 °C for 15 min. Helium was used as the carrier gas at a constant flow of 1 mL/min. The data was analyzed using a MassHunter Workstation B.06.00 SP1, with identification of the individual components performed by comparison with the co-injected pure compounds and by matching the MS fragmentation patterns and retention indices with literature data or commercial mass spectral libraries (NIST/EPA/NIH 2008; HP1607 purchased from Agilent Technologies, Palo Alto, CA, USA).

#### 2.3.2. GC-FID Analysis of TC Essential Oil

The GC-FID analysis of a TC essential oil solution in hexane (dilution ratio 1:200 *v*/*v*, injection volume 1 μL) was carried out by using an Agilent 4890N (Palo Alto, CA, USA) equipped with a FID and an HP-5 capillary column (30 m × 0.25 mm, film thickness of 0.17 μm). The column temperature was held at 60 °C for 3 min, then increased to 210 °C at a rate of 4 °C/min and held at 210 °C for 15 min, then increased to 300 °C at a rate of 10 °C/min, and finally held at 300 °C for 15 min. Injector and detector temperatures were 250 °C. Helium was used as the carrier gas at a flow rate of 1 mL/min. The compound quantification in the essential oil was carried out by the internal normalization of FID chromatogram. Results were expressed as a relative percentage.

A hydrocarbon mixture of n-alkanes (C_9_–C_22_) was analyzed separately under the same chromatographic conditions to calculate the retention indexes with the generalized equation by Van del Dool and Kartz [26], Ix = 100[(*t*_x_ − t_n_)/(*t*_n+1_ – *t*_n_) + *n*], where *t*_n_ and *t*_n+1_ are the retention times of the reference n-alkane hydrocarbons eluting immediately before and after chemical compound “X”; *t*_x_ is the retention time of compound “X”.

#### 2.3.3. Targeted LC-MS/MS Analysis of CLP Extract

Liquid chromatography was performed by using a Flexar UHPLC AS system (Perkin-Elmer, Shelton, CT, USA) equipped with a degasser, Flexar FX-10 pump, autosampler, and PE 200 column Oven interfaced to an AB Sciex (Foster City, CA, USA) API4000 Q-Trap instrument as in Manconi et al. [23]. Briefly, a CLP extract solution in methanol (dilution ratio 1:100 *w*/*v*) was filtered through 0.20 μm syringe PVDF filters (Whatmann International Ltd., Little Chalfont, Buckinghamshire, UK) and injected (5 μL) in the LC-MS/MS system. The chromatographic separation was carried out on a XSelect HSS C18 column (Waters, Milford, MA, USA) (100 × 2.1 mm i.d., 2.5 μm d). Mobile phase A was water containing 0.1% formic acid, while mobile phase B was acetonitrile containing 0.1% formic acid. Elution was carried out at 41 °C according to the following flow and solvent gradient: 0–4 min, isocratic 0% solvent B and the flow changes from 300 μL to 350 μL; 4–6 min, linear gradient 0–12% B and the flow achieves 400 μL/min; 6–12 min, linear gradient 12–20% B and flow constant at 400 μL/min; 16–17 min, linear gradient 20–100% B and flow retrieves to 300 μL/min.

All quantitative data were elaborated with the aid of the Analyst software. Results were expressed as μg/mg of the freeze-dried extract.

### 2.4. Vesicle Preparation and Characterization

Soybean lecithin was purchased from Galeno (Potenza, Italy). Glycerol, propylene glycol, and all other products were purchased from Sigma-Aldrich (Milan, Italy).

#### 2.4.1. Vesicle Preparation

The TC essential oil or CLP extract were incorporated into liposomes, glycerosomes, and propylen glycol-penetration enhancer containing vesicles (PG-PEVs) using an environmentally-friendly technique avoiding the use of organic solvents (Table 1).

Liposomes were formulated by dispersing lecithin (60 mg/mL) and TC essential oil or CLP extract (10 mg/mL) in bidistilled water. The dispersions were sonicated (30 cycles, 5 s ON and 2 s OFF; 13 microns of probe amplitude) by using a Soniprep 150 (MSE Crowley, London, UK).

Glycerosomes were formulated by dispersing lecithin (60 mg/mL) and TC essential oil or CLP extract (10 mg/mL) in a water:glycerol blend (50:50 *v*/*v*). The dispersions were sonicated (30 cycles, 5 s ON and 2 s OFF; 13 microns of probe amplitude) by using a Soniprep 150 (MSE Crowley, London, UK).

PG-PEVs were formulated by dispersing lecithin (60 mg/mL) and TC essential oil or CLP extract (10 mg/mL) in a water:propylene glycol blend (50:50 *v*/*v*). The dispersions were sonicated (30 cycles, 5 s ON and 2 s OFF; 13 microns of probe amplitude) by using a Soniprep 150 (MSE Crowley, London, UK).

#### 2.4.2. Vesicle Characterization

The average diameter and polydispersity index (PI; a measure of width of the size distribution) of the vesicles were determined by Photon Correlation Spectroscopy using a Zetasizer nano (Malvern Instruments, Worcestershire, UK). Samples (*n* = 6) were backscattered by a helium-neon laser (633 nm) at an angle of 173° and a constant temperature of 25 °C. The zeta potential of the vesicles, an indicator of the stability of vesicular dispersions, was estimated by using the Zetasizer nano by means of the M3-PALS (phase analysis light scattering) technique, which measures the particle electrophoretic mobility. Prior to the analysis, the samples (10 μL) were diluted with the appropriate mixture (i.e., water or water/glycerol or water/propylene glycol, 10 mL). A long-term stability study was performed by monitoring the vesicle size, polydispersity index, and zeta potential over 60 days at 4 °C.

### 2.5. Cytotoxicity Assay

Human gingival fibroblasts (HGFs) were isolated from biopsies of gingiva from human adult patients, all informed and consenting (ethical approval by ethics committee review boards for human studies in Sassari, ref. number 2034/2014). The samples were disinfected in poly(vinylpyrrolidone)–iodine solution (PVP-I, Sigma Aldrich Chemie GmbH, Munich, Germany) for 3 min and washed twice in Hank’s balanced salt solution (HBSS; Sigma Aldrich Chemie GmbH, Munich, Germany). Dissociation was then performed by mechanical fragmentation and enzymatic digestion in 0.1% type I collagenase (Gibco Life Technologies, Grand Island, NY, USA) for 120 min at 37 °C in Hanks balanced salt solution (HBSS; Sigma Aldrich Chemie GmbH, Munich, Germany). The cell suspension was filtered through a 70 μm cell strainer (Euroclone, Milano, Italy) and centrifuged at 1800 rpm for 10 min. Then, the cells were transferred in culturing medium containing DMEM medium (Invitrogen, Carlsbad, CA, USA) supplemented with 10% heat-inactivated fetal bovine serum (FBS, Invitrogen, Carlsbad, CA, USA), 200 U/mL penicillin, and 0.1 mg/mL streptomycin. The cells seeded in culture flasks were placed at 37 °C in a humidified atmosphere containing 5% CO_2_. The MTT assay was used to analyze the cytotoxic effect of the two extracts on fibroblasts, according to recently published papers [27,28]. In total, 1 × 10^4^ cells were suspended in 96-well plates diluted in 200 μL of medium. After attachment, the cells were incubated for 48 h with 200 μL of medium containing the different compounds to be tested at different concentrations (1, 10, 100 μg/mL). After 48 h, the medium was removed and 100 μL of 0.65 mg/mL MTT were added in each well and incubated for 3 h. The MTT (yellow tetrazolium salt) was enzymatically converted into purple formazan precipitate by viable cells. The concentration of formazan represents an indicator of viable cells. After removing the medium, 100 μL of DMSO was added to dissolve the formazan participates. Finally, absorbance was detected at the 570 nm wavelength by an ELISA plate reader (Gemini EM Microplate Reader; Molecular devices, San Jose, CA, USA). Control untreated cells were incubated for 48 h with the medium alone (100% viability).

The relative viability of the cells, as compared to the control, was calculated using the following formula:

% cell viability = (OD570 of treated cells) × 100%/(OD570 of control cells).

The data were entered using SPSS Version 2.0 (IBM SPSS, 2013). An ANOVA test was used to analyze the data obtained, and the level of significance was set at *p* < 0.001.

### 2.6. Antimicrobial Assays

Antimicrobial analysis was conducted using the following planktonic microorganisms: *S. mutans* (ATCC 35668); *C. albicans* (ATCC 10231).

#### 2.6.1. Preparation of the Microbial Inocula

Stock culture of each tested microorganisms maintained at −20 °C was recovered by subculturing on nutrient agar plates using Mueller Hinton Agar (MHA) (Sigma-Aldrich) with the addition of sheep blood for *S. mutans*, while for *C. albicans*, Sabouraud Destrose (IFU) was used in accordance with the European Committee on Antimicrobial Susceptibility Testing. One or two colonies of the same morphological type were selected from the plates and aseptically transferred into test tubes containing 2 mL of sterile saline by means of a sterile loop. McFarland standard was used as a reference to adjust the turbidity of the bacterial suspensions to the required range for bioassays (1–2 × 10^8^ UFC/mL).

#### 2.6.2. Disc Diffusion Method

The assessment of the antimicrobial activity of the raw extracts and that of the corresponding extracts incorporated in the nanovesicles was carried out using the disc diffusion method [12]. Sterile Whatman filter paper discs of a 6 mm diameter were impregnated with 15 μL of each sample in a sterile biological safety cabinet. The discs were then aseptically placed in the center of inoculated petri plates (9 cm in diameter) uniformly spread with 0.1 mL of the overnight culture of each microorganism. The plates were refrigerated at 4 °C for 2 h to allow the samples to diffuse into the agar medium and then incubated upside down at 37 °C for 48 h. The tests were conducted in triplicate and the measurement of the inhibition zones was read at 24 and 48 h. A paper disc without antimicrobials was used as a positive control for the growth of the microorganisms. Gentamycin (10 mg/disc; Gibco) and Ketoconazole (10 mg/disc; Janssen Pharmaceutics) were used as negative controls for bacterial and fungal strains, respectively. The scale applied for the measurement of the antimicrobial activity as the zone of inhibition was as follows (disc diameter included): Not sensitive (−) for total zone diameters ≤ 12 mm; sensitive (+) for diameters ranging between 12 and 19 mm; extremely sensitive (+++) for zone diameters ≥ 20 mm. The bioassays were conducted in a biological safety cabinet in accordance with the protocols of Clinical and Laboratory Standards Institute (CLSI), formerly National Committee for Clinical Laboratory Standards [29].

#### 2.6.3. Minimum Inhibitory Concentration (MIC), Minimum Bactericidal Concentration (MBC), and Minimum Fungicidal Concentration (MFC)

The MIC, MBC, and MFC of the raw extracts and that of the nanovesicles against *S. mutans* and *C. albicans* were determined using a broth microdilution method according to standard guidelines [30,31]. In 96-well microtiter trays, each sample was subjected to two-fold serial dilutions (2.5–0.078 mg/mL) in MHA or Sabouraud Destrose Liquid Medium for bacteria and yeasts, respectively, and the wells were inoculated with 10^3^ to 10^4^ bacteria or yeast cells from 24 h broth cultures; the plates were then incubated at 37 °C for 24 h. The MIC was defined as the lowest concentration of each sample that completely inhibited microorganisms’ growth. MBCs and MFCs were determined by sub-culturing small aliquots (10 μL) of the suspensions from wells not showing microbial onto solid media (MHA and Sabouraud Dextrose Agar for the bacteria and the yeast, respectively). The MBC and MFC values were defined as the lowest concentrations that produced no colonies on the agar plates.

#### 2.6.4. Microbial Killing Rates by Time-Kill Assay

Time-dependent killing of the raw extracts and the nanovesicles were identified against standardized microbial inocula of *S. mutans* and *C. albicans* in a liquid medium not supporting cell growth [32]. Microorganisms in a logarithmic phase of growth were centrifuged at 2000 rpm for 10 min, washed in phosphate-buffered saline (PBS, Dulbecco A; pH 7.3), and resuspended at the density of 5 × 10^5^ to 1 × 10^6^ colony-forming units (cfu)/mL in appropriate volumes of PBS containing suitable concentrations of each bioactive agent. An inoculum of microorganisms in PBS without any antimicrobial was used as a control and was included in each assay. After the inoculation, the suspensions were incubated at 37 °C. At time zero and at predetermined intervals (1, 2, and 3 h), 0.5 mL of the mixtures were aseptically removed and subjected to serial 10-fold dilution in PBS; aliquots of 1 mL of the appropriate dilutions were then placed in petri dishes (90 mm diameter) and 19 mL of molten MHA or Sabouraud Dextrose were added for *S. mutans* and *C. albicans*, respectively, swirling thoroughly. The plates were then incubated for 48 h at 37 °C; the number of viable microorganisms at each time was evaluated by counting plates with 30 to 300 colonies. These evaluations were done in duplicate.

### 2.7. Transmission Electron Microscopy

The ultramorphology of *S. mutans* and *C. albicans* after contact with the raw extracts and the extract loaded vesicles, using MIC values, was studied using transmission electron microscopy (TEM). Cell suspensions of *S. mutans* and *C. albicans* were aseptically removed from the cultures. Each culture was divided into two groups, one of which was left untreated (control), while the other group (treated) was further subdivided into four subgroups for microorganism species, each of which was treated at the MIC of each bioactive agent as for the time-kill assay. Thereafter, both the subgroups and the controls were incubated for 24 h at 37 °C. Subsequently, all the cell material was harvested by centrifugation and prefixed with a 2.5% glutaraldehyde solution overnight at 4 °C. As in previous studies under TEM [33,34,35], the pellets were washed in cacodylate buffer, pH 7.4, post-fixed in 1% osmium tetroxide for 30 min, and washed twice in cacodylate buffer, dehydrated using ethanol in increasing concentrations (25–100%), embedded in epoxy resin to make the blocks of the cell pellets, cut into ultra-thin sections (80 μm in thickness) using a Diatome diamond knife, and stained with lead citrate and uranyl acetate. Samples were placed on 50 mesh copper grids and observed under TEM (Zeiss 109 EM Turbo, Königsallee, Göttingen, Germany).

## 3. Results

### 3.1. Chemical Composition

After GC-MS analysis, a total of 14 compounds were identified, representing 99% of the whole TC essential oil. The chemical composition was dominated by carvacrol, which represents 90% of the total active ingredients contained in the essential oil (Table 2).

The chemical characterization of CLP extract was carried out by targeted LC-MS/MS analysis. Thirteen compounds belonging to flavanones and phenolic acids were identified and quantified, as previously reported [23]. The main compound detected in the extract was quinic acid with a concentration that matched 220 μg/mg of freeze-dried extract.

### 3.2. Vesicles Characterization

TC essential oil and CLP extract were incorporated in different types of phospholipid vesicles, namely liposomes, glycerosomes, and PG-PEVs (Table 1), aiming at protecting the bioactive components from possible degradation and controlling their release.

The physical characteristics of the different vesicle systems are reported in Table 3. TC essential oil and CLP extract loaded liposomes were around 86 and 137 nm, respectively. The addition of glycerol (glycerosomes) or propylene glycol (PG-PEVs) led to an increase in size, which was more significant when using TC essential oil. Regardless of the composition of the vesicles, the polydispersity index, which is a dimensionless measure of the broadness of the size distribution, was always ≤0.3, thus indicating a homogeneous distribution of the vesicle size in the dispersions. The zeta potential of the vesicles was generally highly negative, predicting a good stability of the vesicle dispersions during storage. Indeed, the long-term stability studies (60 days at 4 °C) showed that the variation in the vesicle size, polydispersity index, and zeta potential were never greater than 10%.

### 3.3. Cytotoxicity Assay

**Figure 1:**
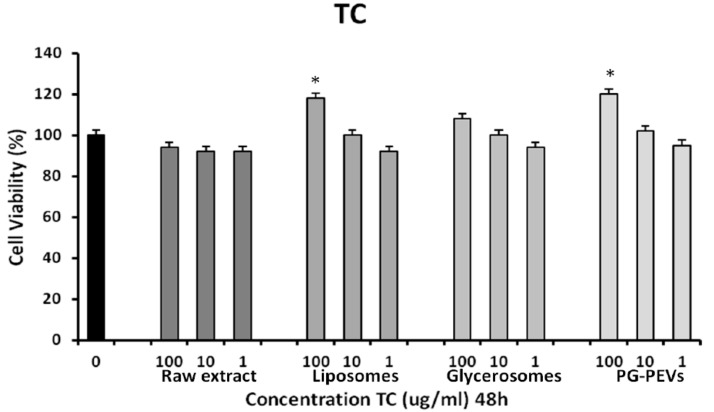
MTT assay after 48 h exposure to different concentrations (100, 10, 1 μg/mL) of TC essential oil, TC liposomes, TC glycerosomes, and TC PG-PEVs. ANOVA analysis showed high significance values (* *p* < 0.001) for liposomes and PG-PEVs at 100 μg/mL vs. the untreated control (0; 100% viability).

**Figure 2:**
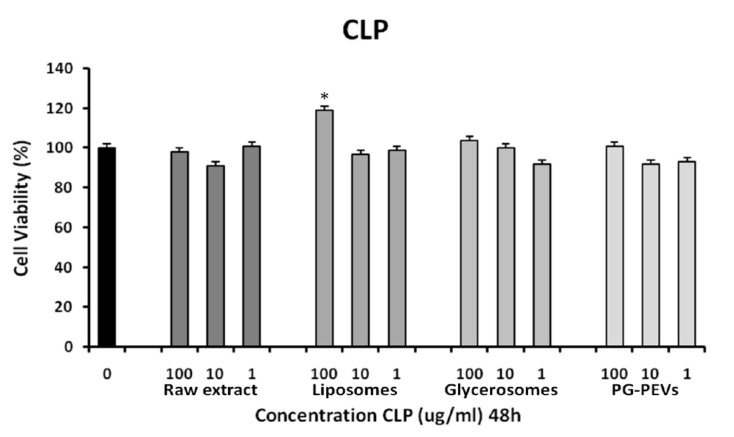
MTT assay after 48 h exposure to different concentrations (100, 10, 1 μg/mL) of CLP essential oil, TC liposomes, TC glycerosomes, and TC PG-PEVs. ANOVA analysis showed high significance values (* *p* < 0.001) for liposomes at 100 μg/mL vs. the untreated control (0; 100% viability).

HGFs were treated or not (control) with different concentrations of TC and CLP for 48 h. The cell viability in the presence of the investigated compounds was assayed. Using one-way ANOVA, no significant differences (*p* < 0.001) were found between the treated cells and control cells. In addition, both TC liposome (100 μg/mL) and TC PG-PEVs (100 μg/mL), as well as CLP liposome (100 μg/mL) treated cells exhibited a significant increase in cell viability, as compared to untreated cells (Figure 1 and Figure 2), indicative of a proliferative effect.

### 3.4. Disc Diffusion Method

Antimicrobial susceptibility tests on discs impregnated with 15 μL of each sample showed that TC had an inhibition capacity towards the tested microorganisms. Also, this preliminary screening demonstrated that TC in the nanovesicles had similar antibacterial effects to the raw essential oil. The raw CLP extract did not display any antimicrobial activity, neither against *S. mutans* nor *C. albicans*. Conversely, when CLP extract was delivered by the nanovesicles, the antimicrobial activity against *S. mutans* increased, while it remained ineffective against *C. albicans*. In Table 4 and Table 5, the antimicrobial activity and 95% CI of the halo inhibition diameters (mm) against *S. mutans* and *C. albicans* are reported.

### 3.5. MIC-MBC/MFC

Raw TC essential oil showed high activity against *S. mutans* with an MIC and MBC of 0.5 mg/mL (Table 6). Raw CLP extract inhibited *S. mutans* with an MIC and MBC of 0.625 mg/mL. However, TC essential oil and CLP extract loaded in liposomes were not effective against the bacteria with an MIC range >2.5 mg/mL, whereas MICs for TC essential oil and CLP extract loaded in glycerosomes and PG-PEVs were <0.078 mg/mL, meaning that no bacterial growth was achieved even at the lower concentrations. Regarding *C. albicans*, MIC and MFC values for the raw TC essential oil were the most effective among the samples tested (Table 7). TC essential oil loaded vesicles were not effective against the yeast (MIC and MBC >2.5 mg/mL), regardless of how they were formulated. MIC and MFC values for raw CLP against *C. albicans* were 0.625 mg/mL, while all vesicles loading CLP extract were not able to inhibit the yeast.

### 3.6. Time-Kill Assay

**Figure 3:**
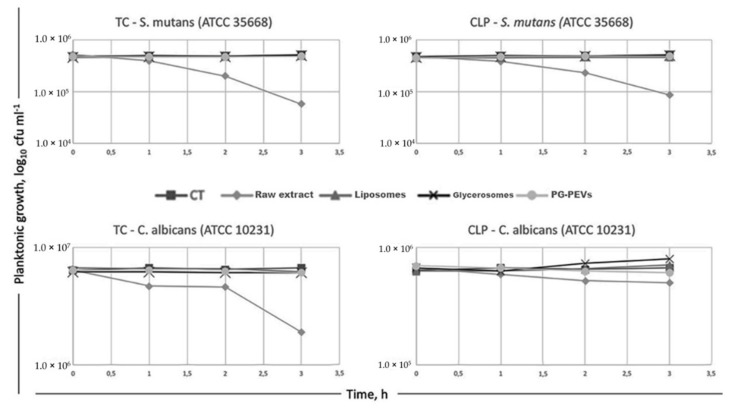
Time-kill curves of *S. mutans* and *C. albicans* treated with TC essential oil, CLP extract, and their nanovesicles.

A time-kill assay was used to further evaluate the antimicrobial activities of the samples. Despite its subjective character, the MIC assay is used to judge the performance of the other methods of susceptibility testing [36]. In our case, MICs for raw TC essential oil and loaded in nanovesicles against *S. mutans* ranged from <0.078 mg/mL to >2.5 mg/mL, while MICs against *C. albicans* ranged from 0.25 mg/mL to >2.5 g/mL. Thus, because of the wide range of MICs, we identified the MICs of raw TC against *S. mutans* (0.5 mg/mL) and *C. albicans* (0.25 mg/mL) as suitable concentrations to conduct a time-course of microbial viability for TC essential oil loaded in vesicles (Figure 3). Similarly, microbial viability for non-incorporated CLP extract and that loaded in vesicles against the target microorganisms was conducted as MICs for the bacteria and the yeast (0.625 mg/mL). The results demonstrated that the inoculum of *S. mutans* was reduced to 11% within 3 h after the addition of raw TC essential oil; and *C. albicans*’ viability was inhibited to 30% after 3 h of exposure to the raw oil. Raw CLP extract reduced the viability of *S. mutans* to 18%, whereas it was not able to inhibit the yeast significantly within the 3 h of this experimentation. The nanovesicles, regardless of the loaded TC essential oil or CLP extract, at the tested concentrations of MICs could arrest neither the growth of *S. mutans* nor that of *C. albicans*, except for CLP in PG-PEVs, which showed an inhibition ability towards the yeast (Figure 3).

### 3.7. Transmission Electron Microscopy (TEM)

#### 3.7.1. Untreated *S. mutans*

**Figure 4:**
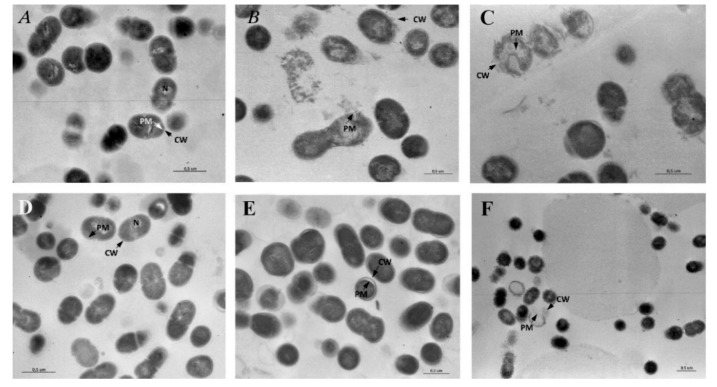
(**A**) Untreated S. mutans shows an intact and well-defined cell wall (CW), plasma membrane (PM), and cytoplasmic space with a nucleoid (N). (**B**) When treated with TC essential oil, S. mutans appears swollen and degenerated with clear evidence of rupture of the CW and PM up to cell disintegration. (**C**) After contact with CLP extract, S. mutans displays features of degeneration comparable to those described in the cells treated with TC essential oil.(**D**) Regardless of the extract loaded, after contact with liposomes, S. mutans showed a well-conserved morphology in comparison to the control, whereas (**E**) after contact with glycerosomes, the cells appear swollen and show the enlargement of the wideness between CW and PM, and (**F**) after contact with PG-PEVs, the cells are surrounded by dense globules infiltrating the CW with perforation of the CW and PM. Other cells appear clearly disintegrated in the cluster.

Under TEM, untreated *S. mutans* appeared in clusters and showed an intact and well-defined cell wall (CW), plasma membrane (PM), and cytoplasmic space. The nucleoid could be seen in some cells located in an eccentric position in the cytoplasm (Figure 4A).

#### 3.7.2. Treated *S. mutans*

When treated with TC essential oil, the cluster of *S. mutans* showed a high range of degenerated cells. The cells appeared swollen and deformed with a clearly observed rupture of CW and PM in addition to cytoplasm degeneration up to cell disintegration (Figure 4B). After contact with CLP extract, *S. mutans* showed morphological aspects of degeneration comparable to those described in the cells treated with TC essential oil (Figure 4C). After contact with liposomes (Figure 4D), *S. mutans* showed a well-conserved morphology in comparison to the control, whereas after contact with glicerosomes (Figure 4E), the cells showed characteristic enlargement of the wideness between the CW and PM, and after PG-PEV contact (Figure 4F), the cells appeared surrounded by dense globules infiltrating the CW and displayed perforation of the CW and membrane. Occasional aspects of cell degeneration were also reported using PG-PEVs.

#### 3.7.3. Untreated *C. albicans*

**Figure 5:**
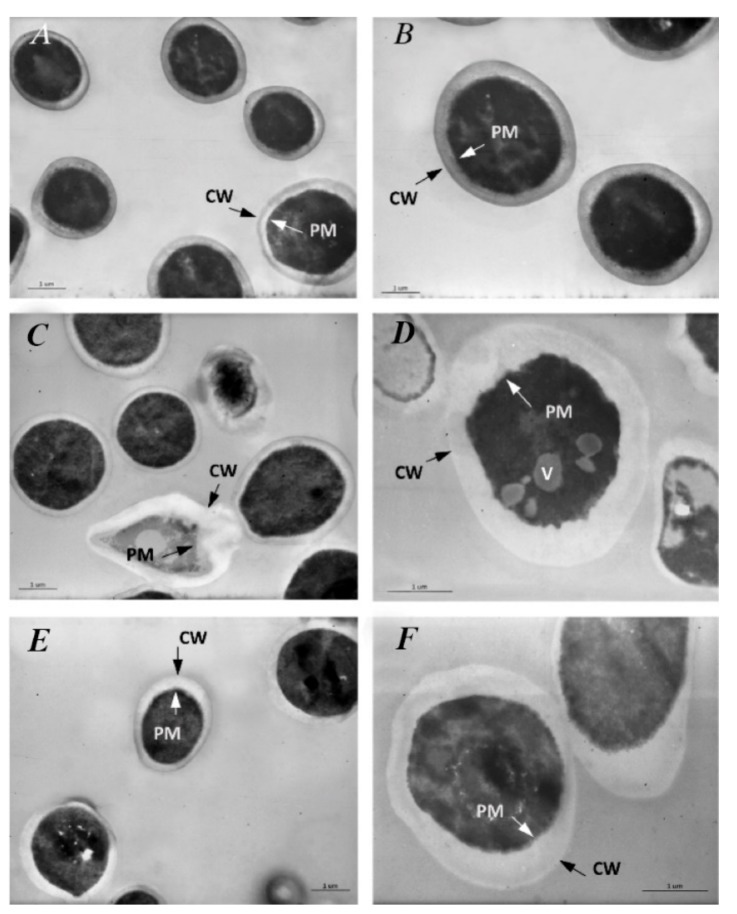
(**A**) Untreated *C. albicans* shows a prominent and amorphous CW surrounding the PM and the dark intracytoplasmatic space. (**B**) Characteristic microfibrils of β glucans can be observed in the outer layer of the membrane. Microfibrils are intermeshed into thicker bundles, forming a wide-meshed network of fibrils extending into the cell wall. (**C**) After 24 h of contact with TC essential oil, the yeast show features compatible with a fungicidal activity. The photograph shows alteration in the shape and wideness of the CW with rupture of the PM and cytoplasm fragmentation of the yeast. (**D**) In other cells, the PM appears ruffled and vacuoles of medium density and shape could be detected within the cytoplasm. (**E**) Protoplasms treated with CLP extract show an enlargement of the wideness between the CW and PM. (**F**) No morphological alterations could be observed neither in the PM nor in the cytoplasm, suggesting a fungistatic rather than a fungicidal effect of CPL extract.

The ultrastructure of the yeast shows a prominent and amorphous CW surrounding the inner PM and the dark intracytoplasmatic space (Figure 5A). Microfibrils of β glucans could be seen in the outer layer of the membrane. Microfibrils formed a wide-meshed network of fibrils extending into the cell wall (Figure 5B).

#### 3.7.4. Treated *C. albicans*

**Figure 6:**
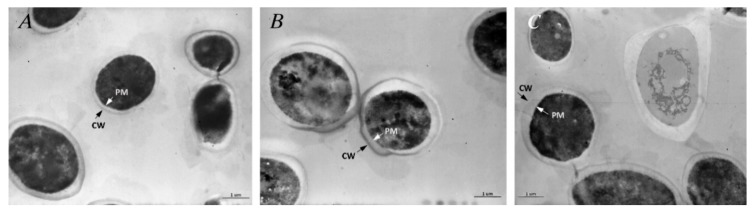
(**A**) After contact with liposomes, the cells show quite conserved morphology in comparison to the control, while (**B**) after contact with glycerosomes and (**C**) PG-PEVs, halos of different thicknesses delimiting and infiltrating the CW are common features in the cells, with some occasional aspects of cell degeneration using PG-PEVs.

After 24 h contact with TC essential oil, *C. albicans* showed an alteration in the shape and wideness of the CW with rupture of the PM together with cytoplasm fragmentation and features compatible with cell death (Figure 5C). Other cells showed an irregularly ruffled PM with vacuoles (V) of medium density and shape in the cytoplasm (Figure 5D). Protoplasms treated with CLP extract showed an enlargement of the wideness between the CW and PM (Figure 5E). However, no morphological alterations could be observed, neither in the PM nor in the cytoplasm, suggesting a fungistatic rather than a fungicidal effect of CPL towards *C. albicans* (Figure 5F). After contact with liposomes (Figure 6A), the cells showed a quite conserved morphology in comparison to the control, while after contact with glycerosomes (Figure 6B) and PG-PEVs (Figure 6C), halos of different thicknesses delimiting and infiltrating the CW were commonly observed in addition to occasional aspects of cell degeneration using PG-PEVs.

## 4. Discussion

Despite developments in the understanding of biological and physico-chemical mechanisms, dental caries remains one of the most common diseases of our era, affecting 60% to 90% of children and 100% of adults worldwide [37]. The main methods of prevention have been based on the daily use of fluoride toothpaste, a reduction in both the amount and regularity of sugar intake, and drinking fluoridated water [6,38,39]. However, the effect of fluorine is mainly to increase resistance to demineralization and promote remineralization in teeth, while its antimicrobial activity has not generally been considered significant [40,41]. Hence, antimicrobial agents, directly interfering with the metabolism of dental plaque, are required in addition to mechanical cleaning [8,42]. In particular, the poor specificity of chemical agents [43] and global antimicrobial resistance [44,45,46] require new and safe molecules able to target microorganisms selectively [47].

Given this context, we chose to study the antimicrobial capacity of TC essential oil and CLP extract with the intention of identifying new natural agents taken from Sardinia’s biodiversity, which could be useful against *S. mutans* and *C. albicans*. The extracts were incorporated in different phospholipid nanovesicles with the intent to protect the bioactive molecules from degradation and volatility, while modulating their delivery over time and improving its bioavailability and potency at the target site [48]. Because in previous studies we had found that glycerosomes and PG-PEVs were effective in transporting bioactive polyphenols [22,23], this study had the intention to test the possibility that the same carriers would be useful in delivering antimicrobial TC essential oil and CPL extract. The vesicle systems used in this experiment demonstrated a homogeneous distribution of particle size (PI ≤ 0.3) and a highly negative zeta potential, which, as a result of the index of the magnitude of repulsive interaction between the colloidal particles, ensured the high stability of the nanodispersions [23,49]. Regardless of their antimicrobial aggressiveness, none of the samples examined, at the concentrations used, showed cytotoxicity in gingival fibroblasts. This outcome confirms the use of Thyme biomolecules as safe compounds (GRAS) [50] and contributes to the understanding of Citrus flavonoids in the field of modern medicine. In addition, the data confirm the possible use of phospholipid nanovesicles as oral systems [51].

The data also suggest that at selected doses, there will be a selective cytotoxicity of the natural extracts towards microbes due to the fundamental differences in composition and structure of the host cells, compared to those of bacteria and yeasts [52]. TC essential oil was the most effective antimicrobial. The very high presence of the phenol carvacrol in TC essential oil (90.1%) could explain the major activity of this fraction against *S. mutans* and *C. albicans* [15,17,53,54]. However, a synergistic interaction of the oxigenate, terpene (1.1%); the bicyclic sesquiterpene, caryophyllene oxide (1.3%); the terpenes, p-cymene and terpinen-4-ol (1.1%); and thymol (1.1%) could also be involved in the antimicrobial ability [55,56]. In addition, a direct effect of caryophyllene oxide has been reported against *Streptococcus* [57]. With regards to carvacrol, its high toxicity against microorganisms derives from its hydrophobicity and allows it to partition the lipids of the cell membrane [58], making the membrane destabilized and permeable [53,59,60], and thus leading to the leakage of ions and other cell constituents, the impairment of several enzyme systems [17,61]**,** and also endoplasmic reticulum stress [15]. Toxicity requires a pH < pKa, in which case the peptide is fully positively charged, capable of interacting with the anionic membrane of the cells [58], and thus able to enter the phospholipid bilayer of the membrane. Accordingly, we used culture media with a pH range of 5.7–7 ± 0.1, which allowed the pH dependence of carvacrol [53,59], pKa phenolic OH group ∼10.9 [62]. *S. mutans* and *C. albicans* were severely inhibited by the raw essential oil as shown by the MBC-MFC/MIC ratio and the time-kill kinetics (Table 6 and Table 7, Figure 3). In particular, the MIC and MFC, in addition to the killing kinetics, demonstrated that the TC essential oil was the most effective fungicidal, killing up to 70% of the yeasts and not just arresting their growth. These data were supported by the morphological analysis of *S. mutans* and *C. albicans* showing cell features compatible to lethal and not inhibitory effects.

Regarding CLP extract, the chromatographic technique coupled with mass spectrometry revealed it was rich in flavanones, flavones, and phenolic acid mainly represented by quinic acid (219.7 μg/mg), followed by neoeriocitrin (46.5 μg/mg), neohesperidin (44.6 μg/mg), naringin (23.8 μg/mg), and sinapic acid (30.1 μg/mg) [23]. Although the compounds in CLP extract belong to a different class with respect to the components found in TC essential oil, they exerted the antimicrobial activity in a similar way by reducing the growth and viability of *S. mutans*, as demonstrated by the MIC and time-kill assays. This result is not in agreement with the disc diffusion assay, which seemed to demonstrate that CLP extract was unable to inhibit *S. mutans*. We could explain the data as a misinterpretation of the therapeutic efficacy of the extract using the agar disc. In fact, it is well known that the inhibition halo is an effect of the capacity of the antimicrobial agent to diffuse into the agar, inhibiting germination and growth of the test microorganism. However, it is also possible that antimicrobial cationic agents could combine with the anionic agar polysaccharide gel [63], making this method imprecise in effectively predicting the capacity of a compound. Despite its bactericidal ability, CLP extract was not effective as fungicidal compound, as was evidenced by the MIC and time-kill assay (Table 7 and Figure 3). These data, in addition to the TEM images, could suggest a fungistatic rather than a fungicidal effect of CLP extract towards *C. albicans*. To explain the different susceptibility of the yeast to the extracts, a specific toxicity of some of the TC essential oil’s active fractions, in particular, carvacrol [15,17,53,54], could be supposed. A proteic partner in the phospholipid bilayer of the fungal membrane has been proposed for carvacrol. This interplay is similar to most antifungal therapies, e.g., azoles among others, which lead to a fungistatic effect [64]. In addition, antifungal therapies have side effects in humans and cause an increase in *Candida* resistance [64,65,66,67,68]. Thus, although further studies are necessary, among them an assessment of whether emerging resistance could be developed using multiple exposures to TC essential oil, the fungicidal ability of this natural drug should be considered as a safe alternative to chemicals.

Regarding phospholipid vesicles, TC essential oil and CLP extract loaded in liposomes did not display any antibacterial activity against *S. mutans* (MIC > 2.5 mg/mL). Conversely, TC essential oil and CLP extract loaded in glycerosomes and PG-PEVs resulted in MICs that were unquantifiable, meaning that no bacteria growth was detectable under these experimental conditions, even at the lowest concentrations. Thus, it could be possible that glycerosomes and PG-PEVs exerted their antibacterial activities after the 3 h time limit of the kill assay, at which point the raw extracts were effective against the bacteria. It is likely that in some way, the vesicles might slow the release of the loaded bioactive molecules within that time. In addition, we could suppose that the extract transported by the vesicles was not the only factor in determining the toxicity against *S. mutans*. Under TEM, the prime target of glycerosomes and PG-PEVs seemed to be the bacterial CW, which was surrounded by dense halos with a tendency to reach the bacterial membrane. Although the vesicles may be too large to penetrate the bacterial envelope, it is likely that the co-surfactant molecules partition the lipid compartments of the bacterial cells. This may occur either directly (from the vesicles to the bacteria) or indirectly (through the aqueous phase). As larger, lipophilic co-solvent molecules, such as glycerol and propylenglycol, have a higher affinity for lipidic compartments than smaller, more hydrophilic compounds, they might have contributed to the activity of the system [69]. This consideration may explain the different abilities and morphological features of phospholipid vesicles against *S. mutans*.

Regarding the antimicrobial activity against *C. albicans*, glycerosomes and PG-PEVs displayed a fungistatic activity, highlighting a greater resistance of the yeast membrane in comparison to that of the bacteria.

## 5. Conclusions

On the bases of the data obtained in this study, TC essential oil possesses the highest antimicrobial capacity against *S. mutans* and *C. albicans*. CLP extract showed bactericidal properties against *S. mutans*, but it was not effective as a fungicidal compound. Regardless of the extract loaded, glycerosomes and PG-PEVs behaved similarly against both the bacterium and the yeast.

## Figures and Tables

**Table 1:** Composition of TC essential oil and CLP extract loaded liposomes, glycerosomes, and PG-PEVs.

Sample	Soy Lecithin (mg)	Essential Oil/Extract (mg)	Water (mL)	Glycerol (mL)	PG (mL)
TC liposomes	60	10	1.0	--	--
TC glycerosomes	60	10	0.5	0.5	--
TC PG-PEVs	60	10	0.5	--	0.5
CLP liposomes	60	10	1.0	--	--
CLP glycerosomes	60	10	0.5	0.5	--
CLP PG-PEVs	60	10	0.5	--	0.5

**Table 2:** Main components of TC essential oil as analyzed by GC/GC-MS techniques. Results are expressed as relative percentages obtained by internal normalization of FID chromatogram. RI: experimental retention indexes on HP5 column.

Main Components of TC Essential Oil	A%	RI
*p*-cymene	1.1	1022.9
limonene	0.2	1026.5
1,8-cineole	0.6	1028.8
γ-terpinene	0.4	1066.5
linalool	1.6	1100.9
camphor	0.1	1142.5
borneol	0.7	1165.1
terpinen-4-ol	1.1	1176.9
α-terpineol	0.2	1192.7
Thymol	0.5	1294.6
carvacrol	90.1	1306.5
carvacrol acetate	0.1	1375.3
cariophyllene	1.0	1419.3
cariophyllene oxide	1.3	1586.2

**Table 3:** The main physico-chemical properties of TC and CLP loaded liposomes, glycerosomes, and PG-PEVs are reported: Mean diameter (MD), polydispersity index (PI), and zeta potential (ZP) estimated by dynamic and electrophoretic light scattering. Mean values ± standard deviations were obtained from 8 replicates.

Sample	MD (nm)	PI	ZP (mV)
TC liposomes	86 ± 12	0.25	–72 ± 12
TC glycerosomes	479 ± 40	0.23	–49 ± 3
TC PG-PEVs	337 ± 45	0.24	–50 ± 1
CLP liposomes	137 ± 16	0.26	–43 ± 4
CLP glycerosomes	180 ± 16	0.30	–51 ± 9
CLP PG-PEVs	218 ± 28	0.30	–65 ± 5

**Table 4:** Descriptive statistics for the antimicrobial activity of TC essential oil against the microorganisms using the disc diffusion assay.

TC Essential Oil	*S. mutans*		*C. albicans*	
	Mean ± SD	95%CI	Mean ± SD	95%CI
Raw oil	12 ± 0	12–12	12 ± 0.5	10.76–13.24
Liposomes	13 ± 0	13–13	0 ± 0	0–0
Glycerosomes	11 ± 0	11–11	11.83 ± 0.29	11.12–12.6
PG-PEVs	0 ± 0	0–0	9 ± 1.73	4.69–13.30
Gentamicin	14 ± 0	14–14	-	-
Ketoconazole	-	-	11.33 ± 0.58	9.89–12.77

**Table 5:** Descriptive statistics for the antimicrobial activity of CLP extract against the microorganisms using the Disc Diffusion assay.

CLP Extract	*S. mutans*		*C. albicans*	
	Mean ± SD	95%CI	Mean ± SD	95%CI
Raw extract	0 ± 0	0–0	0 ± 0	0–0
Liposomes	8.97 ± 0.58	8.82–9.11	0 ± 0	0–0
Glycerosomes	14 ± 0	14.0–14.0	0 ± 0	0–0
PG-PEVs	10 ± 0	10–10	0 ± 0	0–0

**Table 6:** Minimum inhibitory concentrations (MICs), minimum bactericidal concentrations (MBCs), and minimum fungicidal concentrations (MFCs) (expressed as mg/mL) of TC essential oil and TC loaded in nanovesicles against *S. mutans* and *C. albicans* determined by the broth microdilution method.

Strain	Raw Essential Oil		Liposomes		Glycerosomes		PG-PEVs	
	MIC	MBC/MFC	MIC	MBC/MFC	MIC	MBC/MFC	MIC	MBC/MFC
*S. mutans*	0.5	0.5	>2.5	>2.5	<0.078	<0.078	<0.078	<0.078
*C. albicans*	0.25	0.5	>2.5	>2.5	>2.5	>2.5	>2.5	>2.5

**Table 7:** Minimum inhibitory concentrations (MICs), minimum bactericidal concentrations (MBCs), and minimum fungicidal concentrations (MFCs) (expressed as mg/mL) of CLP extract and CLP loaded in nanovesicles against *S. mutans* and *C. albicans* determined by the broth microdilution method.

Strain	Raw Essential Oil		Liposomes		Glycerosomes		PG-PEVs	
	MIC	MBC/MFC	MIC	MBC/MFC	MIC	MBC/MFC	MIC	MBC/MFC
*S. mutans*	0.625	0.625	>2.5	>2.5	<0.078	<0.078	<0.078	<0.078
*C. albicans*	0.625	0.625	>2.5	>2.5	>2.5	>2.5	>2.5	>2.5
